# Inhibition of sestrin 1 alleviates polycystic ovary syndrome by decreasing autophagy

**DOI:** 10.18632/aging.202872

**Published:** 2021-04-22

**Authors:** Xiaoyuan Xu, Xinli Song, Xiaohua Xu, Yanluan Zheng, Lan Xu, Ling Shen

**Affiliations:** 1 Department of Obstetrics and Gynecology, The First Affiliated Hospital of Shantou University Medical College, Shantou, Guangdong 515041, China; 2 Department of Cardiology, The First People's Hospital of Jingdezhen, Jingdezhen, Jiangxi 333000, China; 3 Department of Cytogenetics Laboratory, The First Affiliated Hospital of Shantou University Medical College, Shantou, Guangdong 515041, China; 4 Department of Microbiology and Immunology, Shantou University Medical College, Shantou, Guangdong 515041, China

**Keywords:** sestrin 1, polycystic ovary syndrome, p53, autophagy

## Abstract

Polycystic ovary syndrome (PCOS) is the most common endocrine disorder in women of reproductive age, accounting for 50–70% of anovulatory infertility cases. However, the etiology of PCOS at the molecular level remains unclear. Here, bioinformatics analysis was performed to identify differentially expressed genes (DEGs) between adipose tissue of PCOS patients and matched tissues from non-hyperandrogenic women. RT-qPCR, western blot, cell counting kit-8 (CCK-8), EdU (5-Ethynyl-2'-deoxyuridine) staining, LC3 staining, ROS (reactive oxygen species) detection, and apoptosis assays were conducted to explore the effects of sestrin 1 on KGN human granulosa-like tumor cells. Bioinformatics analysis indicated that DEGs in adipose tissue from PCOS patients were enriched in the p53 signaling pathway. Moreover, sestrin 1 was identified as a major target of the p53 gene. Downregulation of sestrin 1 inhibited proliferation of KGN cells by inhibiting autophagy. Additionally, sestrin 1 downregulation increased ROS generation and promoted apoptosis in KGN cells. By contrast, overexpression of sestrin 1 increased cell viability by increasing autophagy in KGN cells. Together, these results suggest that downregulation of sestrin 1 may be a potential novel treatment strategy for PCOS.

## INTRODUCTION

As the most common endocrine disorder in women of reproductive age, polycystic ovary syndrome (PCOS) affects 8–12% of those women and accounts for 50–70% of cases of anovulatory infertility [1–3]. The signs and symptoms of PCOS are complex and include androgen excess, hyperthecosis and hyperandrogenemia, minimal granulosa cell proliferation, follicle growth arrest at the small antral stage, and chronic anovulation [4, 5]. In addition to infertility, PCOS is associated with metabolic disorders, including cardiovascular diseases, obesity, insulin resistance, and diabetes [6]. Despite accumulating evidence indicating that both environmental and genetic factors contribute to the pathogenesis of PCOS, the etiology of PCOS at the molecular level remains unclear [7].

Granulosa cells (GCs) are essential for the process of folliculogenesis. Normal proliferation of GCs provides the appropriate microenvironment for oocyte maturation, growth initiation, and atresia of follicles [8]. Dysfunctions associated with GCs may lead to aberrant folliculogenesis and can eventually result in anovulatory infertility [9]. Specifically, GC proliferation is increased in patients with PCOS [7]. Methods for inhibiting increased proliferation of GC cells might therefore provide new treatment strategies for PCOS.

Sestrins are a family of highly conserved cytoplasmic proteins that includes sestrin 1, 2, and 3 and which is targeted by the p53 gene [10, 11]. In particular, sestrin 1 regulates cell growth and cell survivability under various cellular stress conditions, such as DNA damage and oxidative stress [10]. Sestrin 1 may also play a regulatory role in several types of diseases, including cardiac hypertrophy and retinal diseases [10, 12]. However, little research has examined the role of sestrin 1 in regulating PCOS.

## RESULTS

### Identification of DEGs in the adipose tissue of women with PCOS

**Figure 1:**
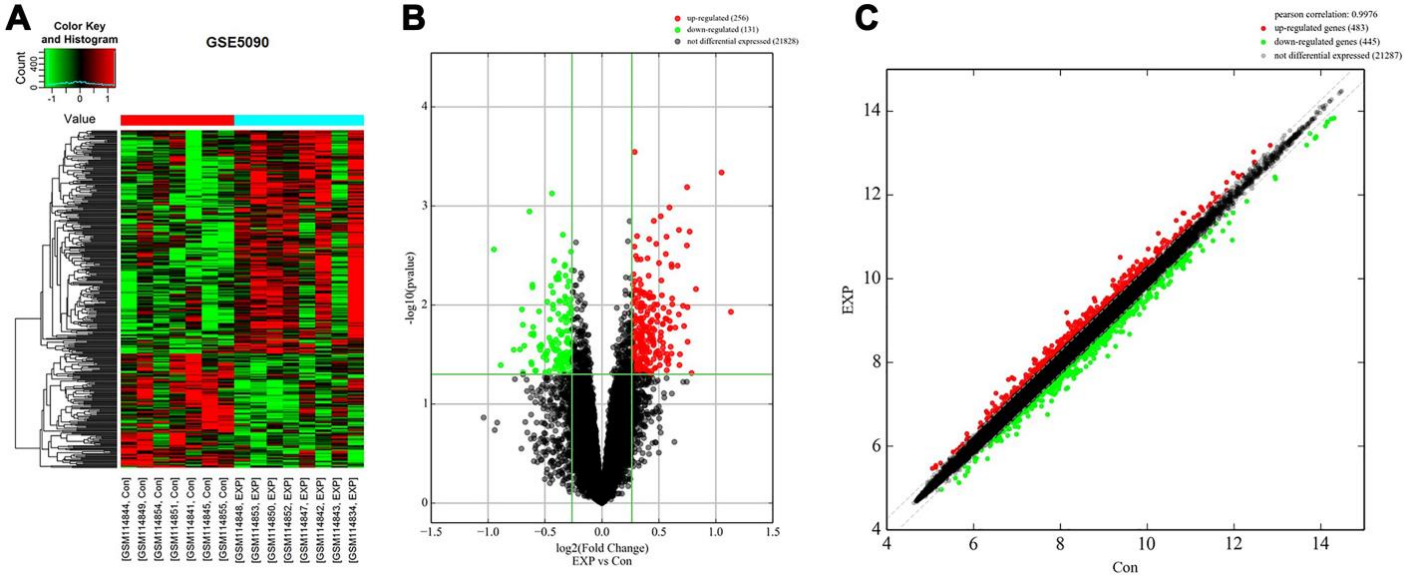
**Identification of DEGs in adipose tissue of PCOS patients.** (**A**) Heat map of DEGs in the GSE5090 dataset. (**B**) Volcano plot of DEGs in GSE5090. Red color indicates upregulated DEGs, and green color indicates downregulated DEGs. *P*-value < 0.05 (-log10 *p*-value > 1.3) and |log2 Fold Change| > 0.25 were used as cutoff values. (**C**) Scatter plot of DEGs in GSE5090.

The expression profile of the GSE5090 dataset was obtained from the Gene Expression Omnibus (GEO) data portal (https://www.ncbi.nlm.nih.gov/geo/). GSE5090 contains expression profiles of omental adipose tissue from 8 PCOS patients and matched tissues from 7 women without PCOS. Differences in DEG expression patterns between PCOS and non-PCOS omental adipose tissue are shown in the heatmap in Figure 1A. DEG expression was also examined in a Volcano plot (cutoff value: *p* < 0.05 (-log10 *p*-value > 1.3) and |log2 Fold Change| > 0.25). As shown in Figure 1B, 256 DEGs were upregulated and 151 were downregulated in adipose tissue from PCOS patients. DEGs showing at least a four-fold change in expression are shown in the scatter plot in Figure 1C.

### Functional and pathway enrichment analysis indicate that sestrin 1 is upregulated in PCOS.

**Figure 2:**
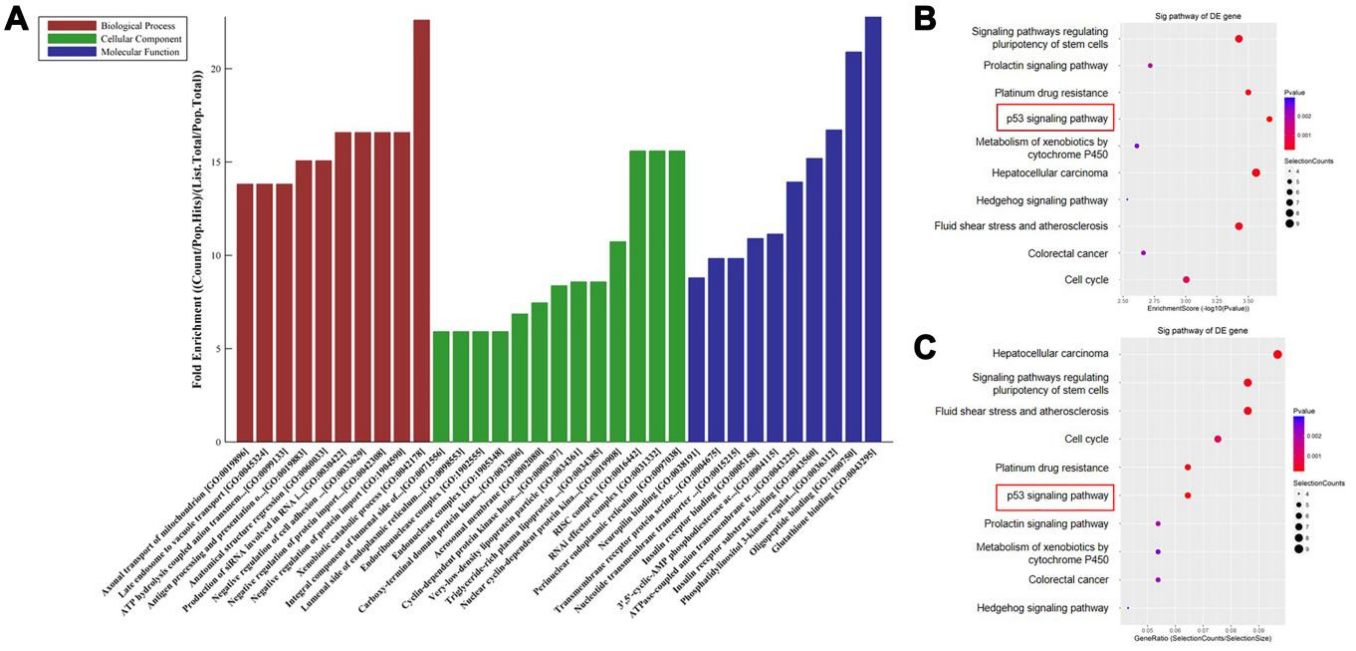
**Functional and pathway enrichment analysis indicate that sestrin 1 was upregulated.** (**A**) The potential functions of DEGs were predicted using GO analysis. (**B**) Scatter plot showing enrichment scores for the 10 most significant enrichment terms. (**C**) Scatter plot showing gene ratios for the 10 most significant enrichment terms. ^**^P < 0.01 compared to the blank control.

Next, GO and KEGG analyses were performed to identify functional processes and pathways in which the DEGs were enriched. The most highly enriched terms in the GO analysis were xenobiotic catabolic process (biological process), perinuclear endoplasmic reticulum (cellular component), and glutathione binding (molecular function) (Figure 2A). The 10 pathways with the highest enrichment scores and the 10 pathways with the highest gene ratio values identified in the KEGG pathway analysis are shown in Figure 2B and 2C. Among the enriched pathways, the p53 signaling pathway is of particular interest given its role in granulosa cells of rats with PCOS [13]. Moreover, the sestrin protein family is targeted by the p53 gene [10, 11]. In addition, the sestrin 1 level was notably upregulated in patients with PCSO, which indicating sestrin 1 might play a role during the progression of PCSO. Thus, the sestrin 1 upregulation identified here was investigated further.

### Downregulation of sestrin 1 inhibited the proliferation of KGN cells

**Figure 3:**
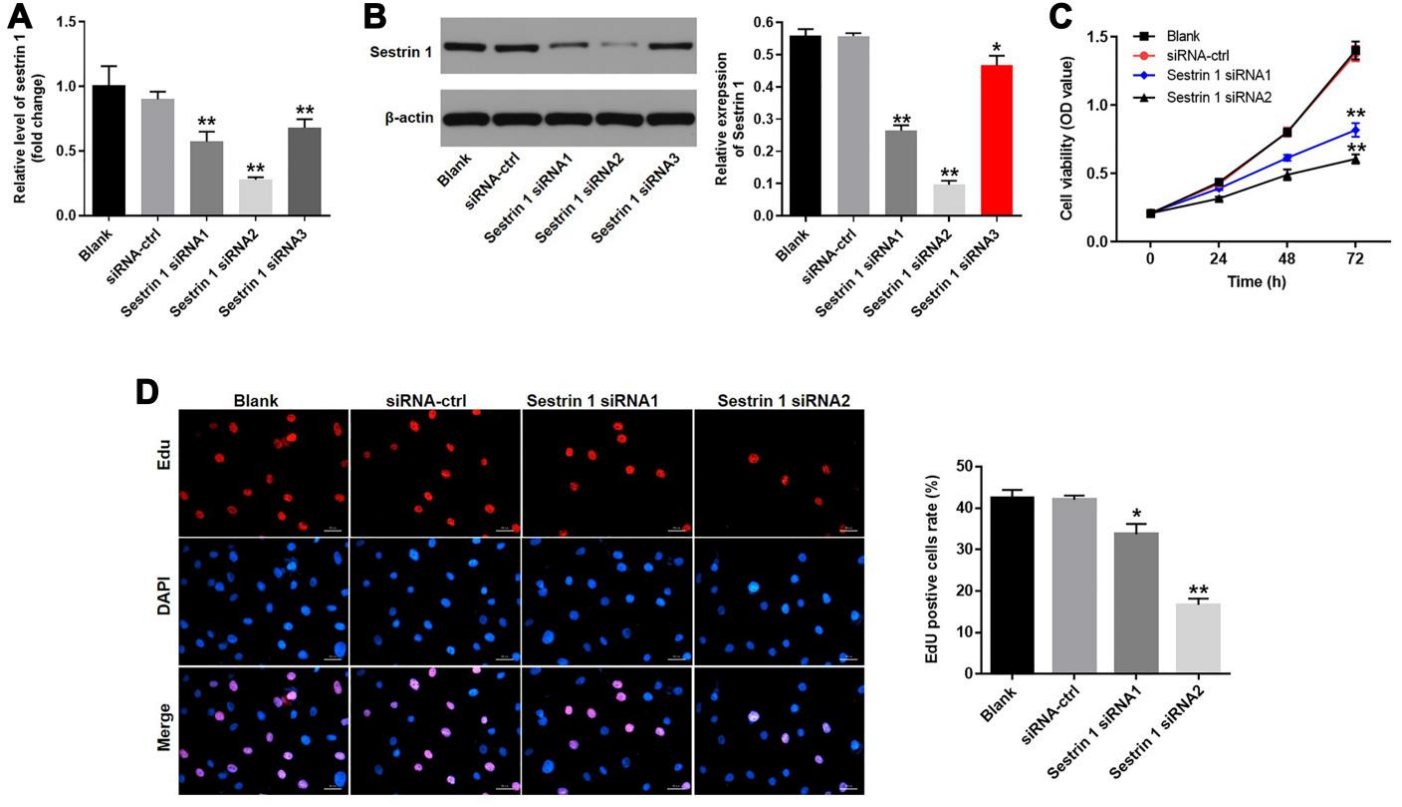
**Downregulation of sestrin 1 inhibited proliferation of KGN cells.** KGN cells were transfected with siRNA control, sestrin 1 siRNA 1, sestrin 1 siRNA2, or sestrin 1 siRNA3 for 48 h. Sestrin 1 silencing efficiency was detected by (**A**) RT-qPCR (**B**) and western blot. The effect of sestrin 1 downregulation on proliferation of KGN cells was determined using (**C**) a CCK-8 assay and (**D**) EdU fluorescence staining. EdU positive cells were counted. ^*^P < 0.05, ^**^P < 0.01 compared to the blank control.

To examine the effects of sestrin 1 in KGN cells, they were transfected with sestrin 1 siRNAs to knock down its expression or siRNA-ctrl. RT-qPCR and western blots indicated that both sestrin 1 siRNA 1 and siRNA 2 were highly effective in silencing expression, confirming the efficiency of transfection (Figure 3A and 3B). In addition, CCK8 and EdU staining were conducted to evaluate the effect of sestrin 1 knockdown on the proliferation of KGN cells. The CCK8 assay showed that downregulation of sestrin 1 markedly decreased the viability of KGN cells (Figure 3C). Similarly, sestrin 1 knockdown notably suppressed proliferation of KGN cells (Figure 3D). Taken together, these results demonstrate that sestrin 1 knockdown significantly suppressed proliferation of KGN cells. Since sestrin 1 siRNA 2 inhibited cell proliferation to a greater extent than siRNA1, sestrin 1 siRNA 2 was used in subsequent experiments.

### Sestrin 1 knockdown inhibited autophagy in KGN cells

**Figure 4:**
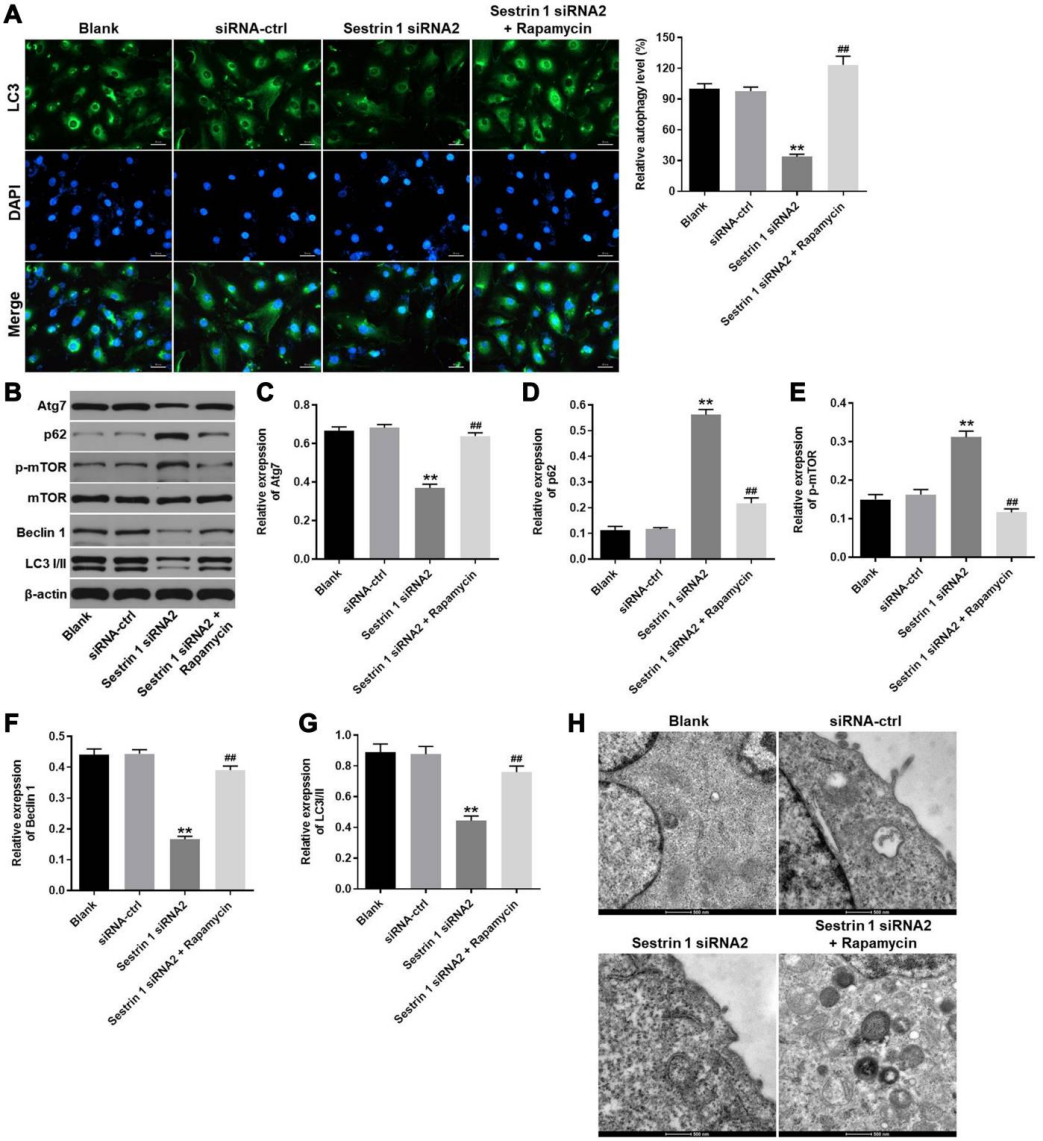
**Sestrin 1 knockdown significantly inhibited autophagy in KGN cells.** KGN cells were transfected with negative control (siRNA-ctrl) or Sestrin 1 siRNA2, and rapamycin was added 48 h after transfection. (**A**) Immunofluorescence staining for LC3 was used to evaluate the effects of sestrin 1 downregulation on autophagy in KGN cells by calculating the percentage of cells that were LC3 positive. (**B**) Western blots were performed to determine levels of Atg7, p62, p-mTOR, mTOR, Beclin 1 and LC3 I/II. β-actin was used as an internal control. (**C**–**G**) Atg7, p62, p-mTOR, Beclin 1 and LC3 I/II expressions were quantified. (**H**) The formation of autophagosome in each group was observed by the electron microscope. ^**^P < 0.01 compared to the blank control. ^##^*P* < 0.01 compared to the sestrin 1 siRNA2 group.

Next, immunofluorescence staining for LC3 was used to explore the effect of sestrin 1 siRNA2 on autophagy in KGN cells. The results indicated that sestrin 1 knockdown significantly inhibited autophagy (Figure 4A). As expected, the autophagy activator rapamycin reversed the inhibitory effect of sestrin 1 siRNA2 on autophagy (Figure 4A). The expression of autophagy-associated proteins, including Atg7, p62, and p-mTOR, was also examined by western blot. Sestrin 1 siRNA 2 markedly decreased Atg7 expression, but increased p62 and p-mTOR levels, in KGN cells (Figure 4B–4G). These changes were also reversed in the presence of the rapamycin (Figure 4B–4G). In addition, The data of TEM suggested there was few autophagosome was observed in sestrin 1 group, while there were some autophagosomes could be found in sestrin 1+rapamycin group (Figure 4H). These findings suggest that sestrin 1 knockdown significantly inhibited autophagy in KGN cells.

### Sestrin 1 knockdown increased oxidative stress in KGN cells by inhibiting autophagy

**Figure 5:**
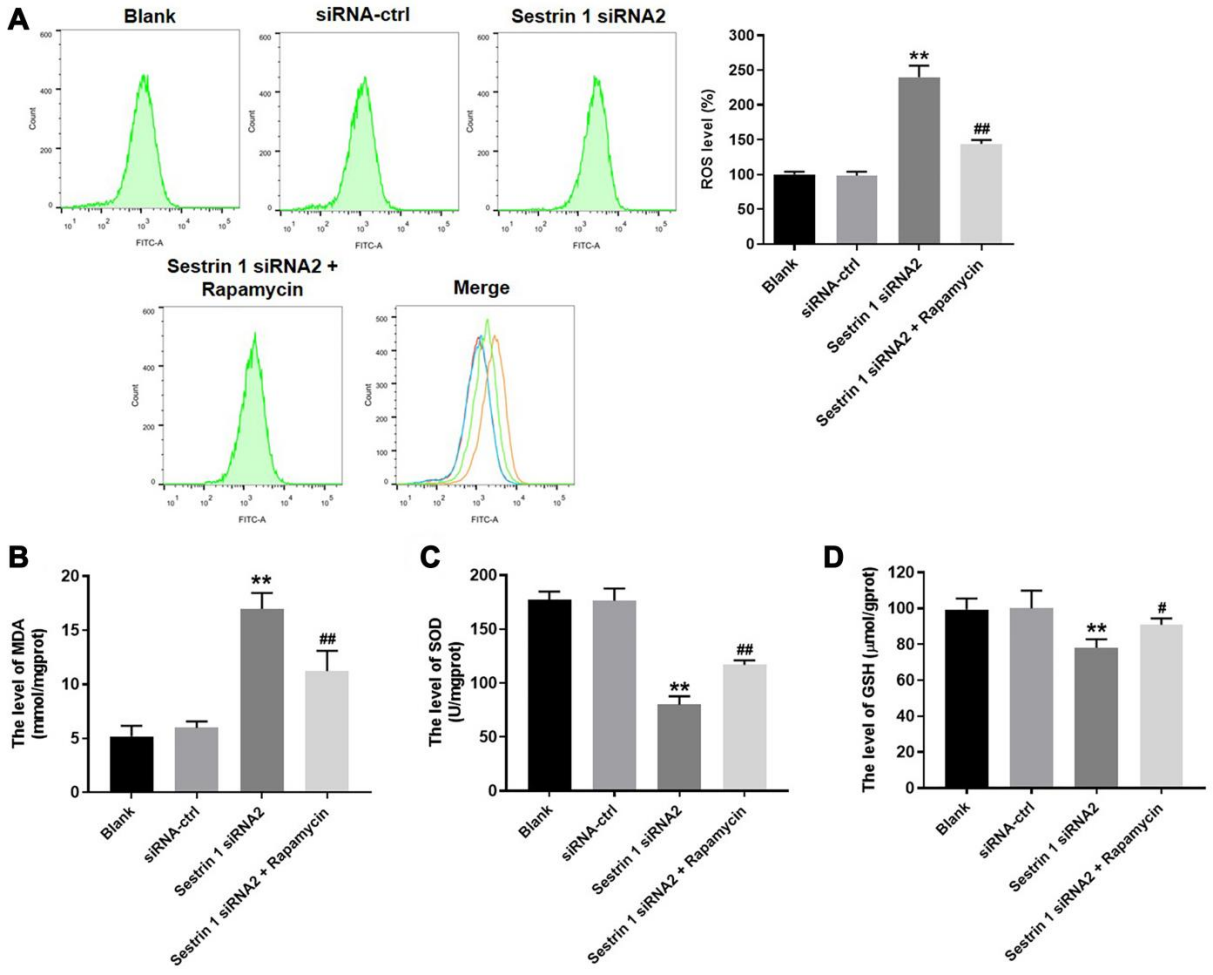
**Sestrin 1 knockdown increased oxidative stress in KGN cells by inhibiting autophagy.** After treatment, (**A**) ROS level in KGN cells were measured using the DCFH-DA method. Results are presented as the percentage of blank control. (**B**–**D**) MDA, SOD, and GSH levels were measured by ELISA. ^**^P < 0.01 compared to the blank control. ^#^*P* < 0.05, ^##^*P* < 0.01 compared to the sestrin 1 siRNA2 group.

We next investigated the levels of ROS, MDA, SOD, and GSH in KGN cells to evaluate the effect of sestrin 1 on oxidative stress. As shown in Figure 5A, ROS generation in KGN cells was notably increased by sestrin 1 siRNA 2, and this effect was reversed by rapamycin. In addition, sestrin 1 knockdown increased MDA levels, but decreased SOD and GSH levels in KGN cells (Figure 5B–5D). Furthermore, these expression changes were reversed in the presence of rapamycin (Figure 5B–5D). Taken together, these results suggest that sestrin 1 knockdown increased oxidative stress in KGN cells by inhibiting autophagy.

### Sestrin 1 knockdown promoted apoptosis in KGN cells by inhibiting autophagy

**Figure 6:**
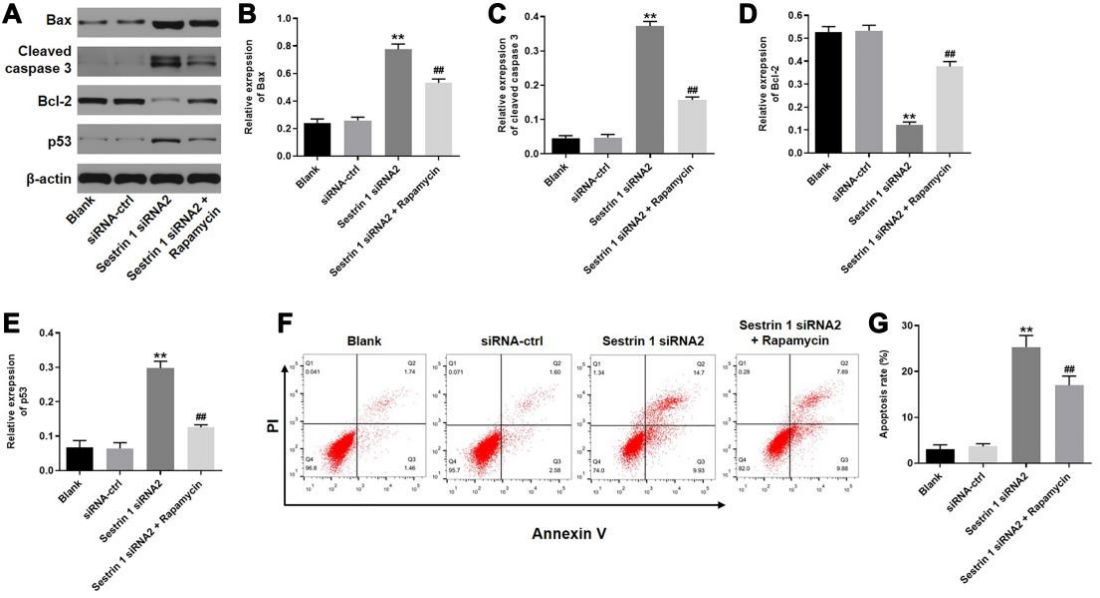
**Sestrin 1 knockdown promoted apoptosis in KGN cells by inhibiting autophagy.** (**A**) Western blots were performed to determine the levels of apoptosis associated proteins, including Bax, cleaved caspase 3, bcl-2, and p53. β-actin was used as an internal control. (**B**–**E**) Quantitative analysis of Bax, cleaved caspase 3, Bcl-2, and p53 expression in KGN cells. (**F**) Cell apoptosis was detected using annexin V/PI staining followed by flow cytometry. (**G**) Cell apoptosis rates were quantified. ^**^P < 0.01 compared to the blank control. ^##^*P* < 0.01 compared to the sestrin 1 siRNA2 group.

To determine the effect of sestrin 1 knockdown on cell apoptosis, Bax, cleaved caspase-3, Bcl-2, and p53 expression were evaluated by western blot. As demonstrated in Figure 6A–6E, sestrin 1 knockdown increased the expression of Bax, caspase-3, and p53, but decreased Bcl-2 expression. In addition, these sestrin 1 siRNA2-induced expression changes were reversed by rapamycin. Moreover, sestrin 1 knockdown increased apoptosis in KGN cells, which was also reversed by rapamycin (Figure 6F–6G). Together, these results suggest that sestrin 1 knockdown promoted apoptosis in KGN cells by inhibiting autophagy.

### Sestrin 1 overexpression promoted KGN cell proliferation by activating autophagy

**Figure 7:**
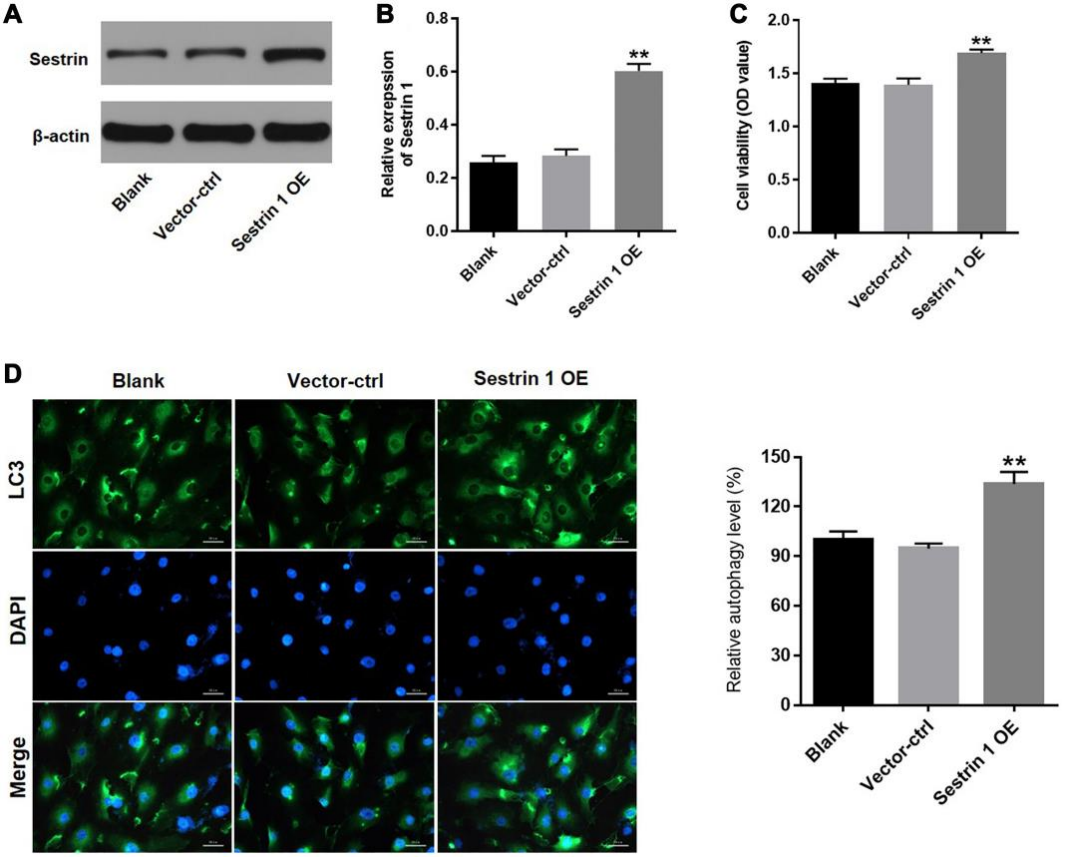
**Sestrin 1 overexpression promoted proliferation of KGN cells by activating autophagy.** KGN cells were transfected with sestrin 1 OE or vector-ctrl for 72 h respectively. (**A**) Sestrin 1 overexpression efficiency was evaluated by western blot. (**B**) Sestrin 1 expression was quantified. (**C**) The effect of sestrin 1 OE on proliferation of KGN cells was determined using a CCK-8 assay. (**D**) The effect of sestrin 1 OE on autophagy in KGN cells was determined using immunofluorescence staining for LC3. The percentage of LC3-positive cells indicates relative levels of autophagy. ^**^P < 0.01 compared to the blank control.

To further explore the role of sestrin 1 in PCOS, sestrin 1 overexpression (OE) KGN cells were established. Western blot data indicated that sestrin 1 was stably overexpressed in KGN cells after transfection (Figure 7A and 7B). In addition, a CCK-8 assay demonstrated that sestrin 1 OE significantly increased proliferation of KGN cells (Figure 7C). Next, LC3 staining was conducted to evaluate the effect of sestrin 1 OE on autophagy in KGN cells. As shown in Figure 7D, sestrin 1 OE markedly increased autophagy. These data suggest that sestrin 1 OE promoted KGN cell proliferation by activating autophagy.

### Sestrin 1 overexpression alleviated the progression of PCOS *in vivo*

**Figure 8:**
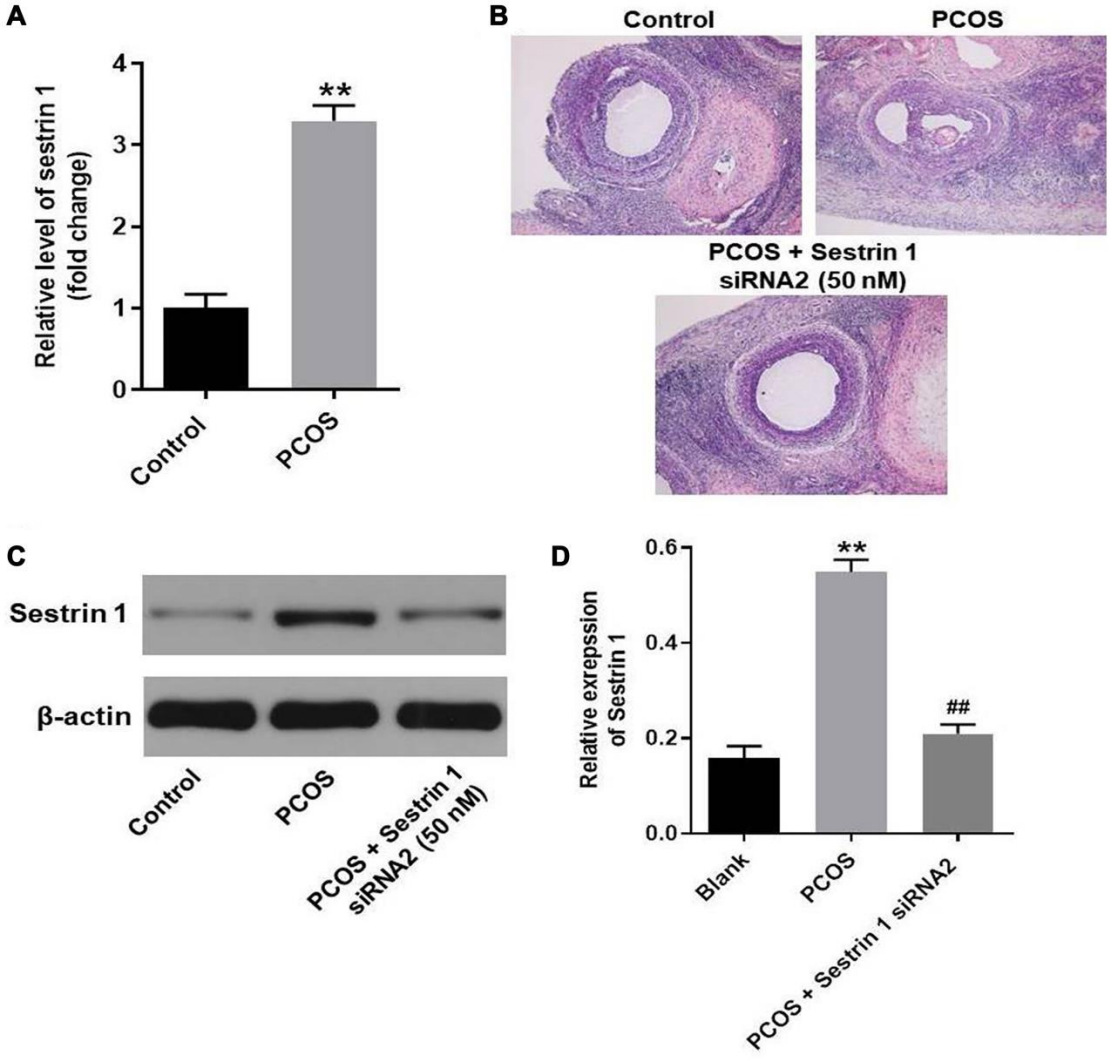
**Sestrin 1 overexpression alleviated the progression of PCOS *in vivo*.** (**A**) Level of sestrin 1 in rat ovarian tissues were detected with RT-qPCR. (**B**) HE staining of ovarian tissues in each group. (**C**) The expression of sestrin 1 was detected with western blot. (**D**) Sestrin 1 expression was quantified. ^**^P < 0.01 compared to the control group, ^##^*P* < 0.01 compared to the sestrin 1 siRNA2 group.

Finally, to explore the function of sestrin 1 in PCOS, PCOS rat model was established. The result of RT-qPCR suggested the level of sestrin 1 was notably upregulated in PCOS rat; this data is consistent with clinical analysis (Figure 8A). In addition, HE staining indicated sestrin 1 overexpression alleviated the progression of PCOS *in vivo* (Figure 8B). Moreover, the data of western blotting suggested sestrin 1 level was significantly upregulated in rat with PCOS; however, the increase of sestrin 1 was completely reversed by sestrin 1 siRNA2 (Figure 8C and 8D).

## DISCUSSION

In this study, we found that sestrin 1 was upregulated in the adipose tissue of PCOS patients. Sestrin 1 knockdown inhibited KGN cell proliferation by inhibiting autophagy, which in turn increased oxidative stress and apoptosis; these changes might inhibit development and progression of PCOS. Consistent with our findings, other studies have described antioxidant effects of sestrins as well as the mechanisms underlying these effects in mammalian cells [14]. In that review, two main pathways that mediate the antioxidant function of sestrins are described. In the first, sestrins promote p62-dependent autophagic degradation of Keap1, upregulate Nrf2 signaling, and consequently increase the expression of antioxidant enzymes [14]. In the second, sestrins blunted mTORC1 activation and thereby reduced the accumulation of reactive oxygen species [14]. Here, we also found that p62 and p-mTOR were involved in the antioxidative effects of sestrin 1 knockdown. However, further research is needed to describe the specific mechanism by which sestrin 1 regulates PCOS.

Sestrin 1 may play a role in the treatment of several types of diseases [10, 15, 16]. However, the specific effect of sestrin 1 varies among different diseases. For example, rosiglitazone inhibited apoptosis in retinal cells in response to oxidative stress by increasing sestrin 1 and SOD-2 expression [12]. In contrast, our findings demonstrated that sestrin 1 knockdown could be a potential treatment strategy for PCOS. Additionally, Xue et al. demonstrated that sestrin 1 was downregulated in hypertrophy, and sestrin 1 overexpression inhibited phenylephrine (PE)-induced cardiac hypertrophy by promoting autophagy [10]. The findings of that study and those of our present study indicate that the effects of sestrin 1 on some diseases are a result of its ability to regulate autophagy. However, sestrin 1-dependent autophagy had opposite effects in hypertrophy and PCOS. Furthermore, opposing effects of sestrin 1 have been described within a single disease type; Ding et al. reported that sestrins might play opposite roles in the regulation of early and late stages of lung cancer [15]. These differences in the regulatory effects of sestrin 1 might be a result of the complexity of disease mechanisms, and further research of the mechanisms underlying the effects of sestrin 1 is therefore needed.

In conclusion, we found in the present study that sestrin 1 knockdown inhibited proliferation, increased oxidative stress, and promoted apoptosis in KGN cells by upregulating autophagy. Downregulation of sestrin 1 might therefore act as a potential novel treatment strategy for PCOS.

## MATERIALS AND METHODS

### Clinical specimens

Adipose tissues from 5 patients with PCOS and 5 healthy volunteers were obtained from the First Affiliated Hospital of Shantou University Medical College, between May 2018 and March 2019. Written informed consent was obtained from all participants. Exclusion criteria were as follows: diabetes, pregnancy, adrenal or thyroid dysfunction, hyperprolactinemia at enrollment. This study was approved by the Institutional Ethical Committee of the First Affiliated Hospital of Shantou University Medical College.

### Bioinformatics analysis

The gene expression profile for the GSE5090 dataset was downloaded from the Gene Expression Omnibus (GEO) website (https://www.ncbi.nlm.nih.gov/geo/). Gene expression profiles in omental adipose tissue from eight PCOS patients were compared to those of matched control tissues from seven women without PCOS. Differentially expressed genes (DEGs) between PCOS adipose tissue and normal adipose tissue were analyzed using R statistical software. *Q*-value < 0.05 and FDR > 2 was used as the cutoff criteria to select the differentially expressed genes Gene. Ontology Consortium (GO) enrichment analysis (http://www.geneontology.org/) was used to identify the cellular functions of DEGs. In addition, Kyoto Encyclopedia of Genes and Genomes (KEGG, https://www.genome.jp/kegg/) enrichment analysis was performed to explore biological pathways associated with the DEGs. A count number > 2 and *P* value < 0.05 were used as the cut-off criteria.

### Cell culture

The human granulosa cell-like tumor cell line (KGN) cell line was obtained from the RIKEN Bioresource Center (Tsukuba, Japan). KGN cells were commonly used to mimic PCOS *in vitro*, since the basic characteristics of this cell line could stimulate the phenotype of disease [17–19].The cells were cultured in Dulbecco’s Modified Eagle’s Medium/F-12 media (DMEM/F-12, Thermo Fisher Scientific, Waltham, MA, USA) supplemented with 10% heat-inactivated fetal bovine serum (FBS, GIBCO, Grand Island, NY, USA), 100 U/mL penicillin, and 100 mg/mL streptomycin (Thermo Fisher Scientific) at 37°C in a humidified incubator with 5% CO_2_.

### Cell transfection and autophagy activation

SiRNAs were synthesized by GenePharma Co., Ltd. (Shanghai, China). SiRNA consisting of a scrambled sequence without significant homology to any known mRNA was used as negative control (siRNA-ctrl). SiRNA oligo sequences were as follows: siRNA1 (5′-GCAAATGGATGGGCCGTTA-3′), siRNA2 (5′-CCATAGACCTTGGCTTATT-3′), siRNA3 (5′-GGTTCATGTTAATCTGCT-3′), and siRNA-ctrl (5′CCACAGTTCCGGTTTAATT3′). Cells were seeded in 6-well plates and cultured overnight before transfection, which was conducted with Lipofectamine 3000 reagent (Life Technologies, Grand Island, NY, USA) according to the manufacturer’s protocols. Transfection efficiency was assessed using RT-qPCR 48 h after cell transfection. After that, 0.2 μg/mL of rapamycin (Sigma Aldrich, St Louis, MO, USA) was added followed by a 1 h of incubation.

### Sestrin 1 overexpression

KGN cells were seeded into 6-well plates and cultured overnight before transfection. Sestrin 1 pcDNA3.1 overexpression (Sestrin 1 OE) and control pcDNA3.1 (vector-ctrl) vectors were constructed by GenScript (Nanjing, China), and transfection was performed according to the manufacturer’s protocol.

### Quantitative reverse transcription polymerase chain reaction (RT-qPCR)

Total cellular RNA was extracted using TRIzol reagent (Takara Biotechnology, Dalian, China). cDNA was synthesized with the PrimeScript RT-qPCR Kit (TaKaRa) following manufacturer’s protocols. Real-time qPCR was used to analyze gene expression with SYBR Premix Ex Taq (Takara) on an ABI 7500 Real-time PCR instrument. Actin was used as an internal control. Relative gene expression was quantified using the 2^-ΔΔCt^ method. Primer sequences were as follows: Actin: forward, 5′-GTCCACCGCAAATGCTTCTA-3′; reverse, 5′-TGCTGTCACCTTCACCGTTC-3′; Sestrin 1: forward, 5′-ATACCGAGTCTTCGGATGGG-3′; reverse, 5’-AATCTGCTTGGTCCCTGTCC-3′.

### Western blot assay

Cells were lysed using RIPA buffer containing proteinase inhibitor (Beyotime Institute of Biotechnology, Shanghai, China) according to the manufacturer’s instructions. Protein concentrations were quantified with the BCA kit (Beyotime). Equal amounts of protein were subjected to sodium dodecyl sulfate polyacrylamide gel electrophoresis (10%), transferred onto polyvinylidene fluoride membranes (Merck Millipore, Billerica, MA, USA), and blocked with 5% non-fat milk at room temperature for 1 h. After that, the membranes were incubated with specific primary antibodies (1:1000, Abcam) at 4°C overnight and then incubated with horseradish peroxidase-conjugated secondary antibodies (1: 2000, Abcam, Cambridge, MA, USA) at room temperature for 2 h. Protein signals were detected using chemiluminescence (ECL) Western blotting detection kits (Merck Millipore, Billerica, MA, USA).

### Cell viability assay

Cell viability was detected with a CCK8 assay as per the manufacturer's instructions (Beyotime). Cells (5 × 10^4^) were seeded into 96-well plates and cultured with 10 μL CCK-8 solution for 3 h. Absorbance was measured at 450 nm with a spectrophotometer.

### Fluorescent staining

For 5-ethynyl-2′-deoxyuridine (EdU) fluorescence staining, the Cell-Light EdU DNA cell proliferation kit (RiboBio, Guangzhou, China) was used following the manufacturer′s instructions. For LC3 staining, cell sections were fixed with 4% paraformaldehyde at 4°C for 20 min followed by permeabilization with 0.1% Triton X-100 and blocking with 3 % BSA at room temperature for 1 min. After washing with PBS three times, cells were incubated with the primary antibody against LC3 (1:1000, Abcam) overnight at 4°C and then incubated with secondary antibodies at room temperature for 1 h. Cells were then counterstained with 10 mg/mL DAPI for 30 min to visualize the nuclei. Images were acquired using a fluorescence microscope (Zeiss, Heidenheim, Germany).

### Reactive oxygen species (ROS) detection

A fluorescent staining method using the ROS probe DCFH-DA (Beyotime) was conducted to measure ROS levels. After they were harvested and washed with PBS, cells were seeded into black 96-well plates and incubated with DCFH-DA for 30 min at 37°C. Fluorescence was then detected by flow cytometry. Mean DCFH-DA fluorescence intensity was used to quantify ROS levels.

### ELISA analysis

Levels of superoxide dismutase (SOD), malondialdehyde (MDA), and glutathione (GSH) in cell lysates were determined according to the instructions of the corresponding Kits (Nanjing Jiancheng Bioengineering Institute, Jiangsu, China). Absorbance was measured at 450 nm using a microplate reader (Bio-Rad Laboratories, Hercules, CA, USA).

### Apoptosis assay

After harvesting and centrifugation, cells (1 × 10^5^/mL) were collected in separate tubes. The Annexin V-FITC Apoptosis Detection Kit (Thermo Fisher Scientific) was used to evaluate cell apoptosis in accordance with the manufacturer’s protocols. The cells were subjected to flow cytometry (BD FACSAria; BD Biosciences, Franklin Lakes, NJ, USA) analysis within 1 h.

### Transmission electron microscope

Cells were washed in PBS and fixed in 2.5% glutaraldehyde for 2 h. After that, cells were fixed with 1% osmium tetroxide buffer for 2 h, dehydrated using a graded ethanol series (50%, 70%, 90%, 100% ethanol, 3 min each), and then embedded in 812 resin. Later on, samples were stained with aqueous uranyl acetate and lead citrate. Subsequently, transmission electron microscope (JEM-1400PLUS; JEOL, Tokyo, Japan) was used to observe autophagosomes.

### Animal study

Female SD rats (21 days old) were obtained from Guangdong Medical Laboratory Animal Center (Foshan, Guangdong). All experiments were approved by the Institutional Ethical Committee of the First Affiliated Hospital of Shantou University Medical College and performed in accordance with the Guidelines for the Care and Use of Laboratory Animals. Rats were randomly divided into 3 groups: Control, PCOS and PCOS + Sestrin 1 siRNA2 group. Rats in the PCOS and PCOS + Sestrin 1 siRNA2 groups were subcutaneously injected with DHEA (6 mg/100g body weight dissolved in 0.2 ml of PBS) for 20 consecutive days. Rats in the control group were injected with equal volume of PBS. Meanwhile, rats in the PCOS + Sestrin 1 siRNA2 group were injected intravenously with 50 nM Sestrin 1 siRNA2 once daily. After 20 days of treatment, animals were euthanized, and ovarian tissues were collected. After that, haematoxylin and eosin (H&E) staining assay was performed to observe the histopathological changes in ovarian tissues. Sestrin 1 siRNA2 could be used for target validation studies in animal disease.

### Statistical analysis

All experiments were repeated at least three times. All data are presented as means ± SD. GraphPad Prism (GraphPad Software, CA, USA) was used for statistical analysis. One-way analysis of variance (ANOVA) followed by Tukey’s test was performed to compare difference between groups. P < 0.05 was considered statistically significant.

### Data availability

The datasets generated and/or analyzed during the current study are available from the corresponding author upon reasonable request.
